# Analyzing the Successful Incompetent to Be Executed Cases in the United States: A First Pass

**DOI:** 10.3390/bs15030325

**Published:** 2025-03-06

**Authors:** I-An Su, John H. Blume, Stephen J. Ceci

**Affiliations:** 1 Department of Psychology, Cornell University, Ithaca, NY 14853, USA; jb94@cornell.edu (J.H.B.); sjc9@cornell.edu (S.J.C.); 2 Cornell Law School, Ithaca, NY 14853, USA

**Keywords:** competency to be executed, competency for execution, death penalty, mental illness, legal decision-making, schizophrenia

## Abstract

More than three decades ago, the Supreme Court of the United States (SCOTUS) ruled that individuals who are not competent (alternatively referred to by the Court as insane) at the time of their scheduled execution cannot be put to death. Despite the years that have passed since the Court’s decision and the literal life-or-death stakes involved, competency for execution (CFE) remains underexplored in the psychological, psychiatric, and legal literature. A number of important legal and ethical issues that arise when a person on death row maintains they are not competent to be executed are still unresolved even after the landmark Supreme Court cases such as *Ford v. Wainwright* (1986), *Panetti v. Quarterman* (2007), and *Madison v. Alabama* (2019). In this first-of-its-kind descriptive study, we analyzed the demographic and case characteristics of the 28 successful *Ford* claimants—individuals in the United States who have been found to be incompetent to be executed and compared them to the general death row population and homicide cases nationwide. Our findings reveal some similarities but also some differences between these claimants and the general death row population and homicide cases: the successful *Ford* claimants are exclusively male (in keeping with the general prison population on death row), relatively older, and underrepresented among White and Latinx inmates (i.e., Black claimants are more successful than their White and Latinx counterparts at evading execution). Nearly all (96%) suffer from schizophrenia, with 79% experiencing psychiatric comorbidity, yet only 54% received any significant treatment before or after the criminal offense. The claimants’ cases also involve a higher proportion of child victims, male family members, and female non-family member victims, as well as more multiple-victim cases (not indiscriminate) and fewer intraracial homicides. Fewer victims are male, and more are female. However, the cases do not align with typical male-on-male violent crimes or femicide patterns, such as those involving sexual or domestic violence. Additionally, systematic psycho-legal deficiencies are prevalent, including a low rate of mental health evidence (61%) presented at trials and some cases lacking psychiatric involvement in CFE evaluations. Temporal influence and drastic state variations on CFE evaluation are also noted. Although the small sample size limits generalizability, this small-scale descriptive study offers a number of important insights into the complexities of CFE decisions and lays the groundwork for future research and policy development.

## 1. Introduction

Competency to be executed, also known as competency for execution (CFE), pertains to assessing an individual’s mental suitability for undergoing state-sanctioned homicide. When an attorney for a condemned person on death row raises a CFE claim, and if the individual is deemed incompetent to be executed, their life is spared (at least temporarily). Conversely, if the courts determine the individual is competent to be executed, the execution proceeds. Therefore, the outcomes of CFE evaluations have profound implications, as they are literally a matter of life or death. In this respect, CFE is distinct from other criminal competency determinations, and the high stakes and the legal, moral, and ethical questions that arise when assessing an individual’s capacity for execution, CFE is one of the most critical forms of decision-making.

CFE is also sometimes referred to as the “last competency” (Brodsky et al., 2001), as it typically represents the culmination of criminal competency evaluations. It becomes relevant only when a defendant’s guilt has been established, other appeals have been unsuccessfully pursued, and the execution date has been set. Therefore, CFE is the final criminal competency for which evaluations may be requested, conducted, and adjudicated. In what follows, we examine a data set that, while small, is complete: we have included every successful case decided over the 40 years since *Ford* was decided. Thus, despite being small, it is not an unimportant group of cases for researchers to examine, analyze, and report findings. We do not compare these cases to unsuccessful cases, which is something we might tackle in later research. 

### 1.1. Historical Development of CFE

#### 1.1.1. Common Law and the Eighth Amendment

The English common law tradition, which prohibits the execution of individuals deemed insane, has played a foundational role in shaping modern legal standards regarding CFE. As cited at the beginning of the landmark Supreme Court case *Ford v. Wainwright* (1986), which we will discuss in the next section, Sir William Blackstone stated the following:

[I]diots and lunatics are not chargeable for their own acts, if committed when under these incapacities: no, not even for treason itself. Also, if a man in his sound memory commits a capital offence, and before arraignment for it, he becomes mad, he ought not to be arraigned for it: because he is not able to plead to it with that advice and caution that he ought. And if, after he has pleaded, the prisoner becomes mad, he shall not be tried: for how can he make his defence? If, after he be tried and found guilty, he loses his senses before judgment, judgment shall not be pronounced; and if, after judgment, he becomes of nonsane memory, execution shall be stayed: for peradventure, says the humanity of the English law, had the prisoner been of sound memory, he might have alleged something in stay of judgment or execution.

In the same vein, Sir Edward Coke (1680, as cited in *Ford v. Wainwright*, 1986) similarly articulated one of the earliest rationales for this principle:

[B]y intendment of Law, the execution of the offender is for example,…but so it is not when a mad man is executed, but should be a miserable spectacle, both against Law, and of extream inhumanity and cruelty, and can be no example to others.

The foundational principles stated by Sir Blackstone and Sir Coke highlight the English common law’s long-standing commitment to safeguarding insane individuals from being executed, a principle with deep historical roots and a consistent reputation as a “savage and inhuman” practice (*Ford v. Wainwright*, 1986).

The long-standing common law tradition that has informed much of American law, including provisions such as those reflected in the Eighth Amendment—“Excessive bail shall not be required, nor excessive fines imposed, nor cruel and unusual punishments inflicted”—prohibits the execution of individuals deemed incompetent. While the criterion for CFE can, in some respects, be opaque and also not fully formed, as we will explore in the following section, this criterion has significantly evolved through landmark Supreme Court cases.

#### 1.1.2. Landmark Supreme Court Cases

The “modern” Supreme Court (Perlin & Harmon, 2021) has issued three landmark decisions over the past four decades addressing whether executing the insane violates constitutional protections against cruel and unusual punishment. These cases have incrementally shaped the definition and interpretation of CFE: *Ford v. Wainwright* (1986), *Panetti v. Quarterman* (2007), and *Madison v. Alabama* (2019).

***Ford v. Wainwright* (1986).** *Ford v. Wainwright* (1986) is the groundbreaking Supreme Court case that addressed, for the first time, the issue of whether it is constitutional to execute an inmate who is insane. The case emerged from the conviction of Alvin Bernard Ford, who was found guilty with three other men of the murder of a police officer and sentenced to death in 1974 after an attempted robbery at a Florida Red Lobster restaurant ended in a fatal shooting.

At the time of the offense, trial, and sentencing, Ford exhibited no obvious signs of mental illness. However, during his years on death row, he developed significant psychiatric symptoms. These included pervasive delusions, such as an obsession with conspiracy theories involving the Ku Klux Klan and, later, a belief that he was Pope John Paul III. In 1983, after 14 months of evaluation, he was diagnosed with paranoid schizophrenia with suicide potential.

Ford’s first death warrant was issued in 1981 but was later halted 14 h before the execution. When Ford’s second death warrant was issued in 1984, his attorneys contended that his severe mental symptoms rendered him incompetent for execution. Using procedures subsequently deemed inadequate, the Florida courts determined that he was competent, and the lower federal courts that adjudicated Ford’s CFE claim agreed. His attorneys asked the Supreme Court to hear the case, and they agreed to do so, thus bringing to the Supreme Court the unprecedented issue of whether executing an individual who cannot comprehend their execution is constitutionally permissible.

The Supreme Court, in an opinion authored by Justice Thurgood Marshall, concluded that the Eighth Amendment prohibits the execution of individuals who are insane and that Florida’s CFE procedures were inadequate, citing English common law and, in his own words, concluding the following:

[T]he Eighth Amendment prohibits the State from inflicting the death penalty upon a prisoner who is insane. The reasons at common law for not condoning the execution of the insane—that such an execution has questionable retributive value, presents no example to others, and thus has no deterrence value, and simply offends humanity—have no less logical, moral, and practical force at present. Whether the aim is to protect the condemned from fear and pain without comfort of understanding, or to protect the dignity of society itself from the barbarity of exacting mindless vengeance, the restriction finds enforcement in the Eighth Amendment.

Because four Justices dissented, Justice Lewis F. Powell’s concurring opinion providing the fifth vote for vacating Ford’s death sentence, set the Eighth Amendment substantive standard, which he articulated as follows: “[t]he test for whether a prisoner is insane for Eighth Amendment purposes is whether the prisoner is aware of his impending execution and the reason for it”. Thus, Justice Powell established a two-pronged criterion for CFE: (1) an awareness of the execution and (2) an awareness of the reason for the execution. In the wake of *Ford*, scholars, practitioners, and judges widely discussed and debated this criterion (Dawson & Putnal, 1986; Moore, 1986; Radelet & Miller, 1992).

Justices William H. Rehnquist and Chief Justice Warren E. Burger disagreed, and on this point, they were joined by Justices Sandra Day O’Connor and Byron R. White, concluding that “[t]he Eighth Amendment does not create a substantive right not to be executed while insane” and disagreed with the view that executing the insane should be deemed unconstitutional. However, Justices O’Connor and Justice White did agree with the majority opinion on the issue of due process, stating that Florida’s procedures located the process totally within the Executive Branch and did not allow attorneys for the person whose competency was in question to provide information or challenge the finding of the Governor’s hand-picked CFE examiners violated due process because: “[i]f there is one fundamental requisite’ of due process, it is that an individual is entitled to an ‘opportunity to be heard’”.

While *Ford v. Wainwright* (1986) is obviously important, even seminal, given that it established that executing an individual who is insane violates the Eighth Amendment, it did not provide a precise definition of insanity or resolve Ford’s specific CFE. To the extent a standard for assessing CFE claims emerged, it was contained in Justice Powell’s concurrence. The Court also found Florida’s procedures for resolving CFE claims to be constitutionally deficient for the reasons stated above, but for the most part, the Court left it to the states to develop procedures adequate to protect the new Eighth Amendment prohibition against executing persons who are incompetent to be executed.

Ford’s CFE claim was never resolved as he died in prison of natural causes in 1991 before a final legal determination could be made by the Florida court under the Supreme Court’s new guidelines.

***Panetti v. Quarterman* (2007).** The Supreme Court said no more about CFE for nearly two decades when it granted certiorari in *Panetti v. Quarterman* (2007), which is the next landmark Supreme Court case addressing the execution of the insane.

In 1992, Scott Louis Panetti, who had been hospitalized multiple times for various mental disorders, dressed in camouflage and murdered his estranged wife’s parents in front of his wife and daughter. He then held his wife and daughter hostage for a night before eventually turning himself in.

At his trial in 1995, a court-appointed psychiatrist indicated that Panetti suffered from severe delusions and hallucinations. Despite this, he was deemed competent to stand trial and waive counsel and then represented himself in a “bizarre,” “scary,” and “trance-like” manner (*Panetti v. Quarterman*, 2007), including appearing in court dressed as a cowboy and attempting to subpoena Jesus Christ, the Pope, John F. Kennedy, and other historical figures. Within two weeks, he was sentenced to death by the jury, and in 2003, a death warrant was issued for his execution in 2004. Panetti’s counsel raised the CFE claim, arguing that he was not competent to be executed due to his notable mental illnesses.

Panetti’s case did not fit squarely within the standard established by Justice Powell in his concurring opinion in *Ford v. Wainwright* (1986) discussed above. During the motion hearings in district, appellate, and federal courts, the core question was whether, despite his awareness of his impending execution and the state’s stated reasons for it, Panetti’s delusional belief—that his execution was, in fact, part of a satanic conspiracy to keep him for preaching the gospel rather than a punishment for his crimes—rendered him incompetent for execution. This delusion (a false, fixed belief) led his attorneys to argue that Panetti was incompetent because he lacked a rational understanding of the reason for his execution.

Panetti’s claim was rejected by the state and federal courts based on a narrow reading of Justice Powell’s opinion in *Ford*, but the Supreme Court agreed to hear the case and ruled in a 5–4 decision, rejecting the lower court’s “flawed interpretation”. The majority opinion, penned by Justice Anthony Kennedy, stated that “[a] prisoner’s awareness of the State’s rationale for an execution is not the same as a rational understanding of it,” asserting that mere awareness of the execution and its reasons is insufficient. The Court emphasized that “awareness of a link between a crime and its punishment in a context so far removed from reality that the punishment can serve no proper purpose,” underscoring that executing individuals who do not have this rational understanding fails to meet the objectives of retribution and deterrence that justify the death penalty. Instead, the Court made clear that a rational comprehension of the crime and the relationship between crime and punishment is an essential element of CFE.

*Panetti v. Quarterman* was an important doctrinal clarification as it established that states must evaluate both the awareness and rational understanding of death row inmates regarding their comprehension of the crime and the reasons for their execution. However, like *Ford*, the Supreme Court did not provide a comprehensive Eighth CFE amendment standard of CFE and specifically stated that it was leaving that issue to address at a later time. This lack of clear guidance has led to varying state procedures and criteria for determining CFE. Additionally, as will be discussed later, contrary to the fear expressed by some conservative judges and scholars, *Panetti* did not open the “floodgates” to CFE claims. An empirical study indicates that the ruling has not significantly increased the number of CFE claims (Blume et al., 2014).

After the 2007 landmark Supreme Court decision, Panetti’s case was remanded to a lower federal court, which determined that he was incompetent for execution. In 2014, Texas issued a new death warrant, but the Fifth Circuit Court of Appeals intervened at the last minute to halt the execution. More recently, in 2023, a federal district court ruled that Panetti’s mental disorders, including disorganized thoughts, prevented him from rationally understanding the reason for his execution. As a result, he was found incompetent to be executed for the second time. The Texas Attorney General’s office did not appeal against that ruling.

***Madison v. Alabama* (2019).** Following *Ford v. Wainwright* (1986) and *Panetti v. Quarterman* (2007), *Madison v. Alabama* (2019) is the Supreme Court’s most recent decision addressing the scope of CFE claims, and it is significant because it addressed the issue not in a case involving a person with mental illness but rather a person on death row suffering from dementia.

Vernon Madison had been on death row for over 30 years for the 1985 murder of police officer Julius Schulte during a domestic dispute. Over the decades, Madison’s health deteriorated significantly due to multiple strokes, including major ones in 2015 and 2016, which led to vascular dementia. After the 2016 stroke, his attorneys argued that his severe vascular dementia, which affected both his memory and cognitive functions, impaired his ability to recall committing the murder and rendered him incompetent to be executed under the Eighth Amendment. They contended that, without any memory of the crime, he could not fully comprehend the justification for his execution.

The case had a tortured procedural history in the state and federal courts, but in 2019, the Supreme Court, in a 5–3 decision written by Justice Elena Kagan, reaffirmed that under *Ford* and *Panetti*, the Eighth Amendment prohibits the execution of individuals who lack a rational understanding of their execution and the reasons for it. The Court also clarified that the Eighth Amendment may permit executing a prisoner even if the individual cannot remember the crime or suffers from dementia or another disorder. The key issue, as stated by the Court, is “whether a mental disorder has had a particular *effect*”. “If [memory] loss combines and interacts with other mental shortfalls to deprive a person of the capacity to comprehend why the State is exacting death as a punishment, then the *Panetti* standard will be satisfied”. The Court underscored that memory of the crime, while not an absolute requirement for execution, is a significant factor in assessing whether an inmate can rationally understand the reasons for their punishment. Despite Madison’s awareness of his impending execution and his ability to understand that it was connected to his past actions, his profound cognitive impairments and memory loss raised doubts about his competency under this expanded understanding of rational comprehension.

*Madison v. Alabama* (2019) is significant as it applied CFE to the rapidly aging U.S. death row population (Stanziani et al., 2020) and has significant implications for the evaluations and treatment of chronically incarcerated, aging, and mentally ill prisoners. The case requires courts to consider how conditions like dementia and Alzheimer’s Disease affect a prisoner’s understanding of and ability to process their punishment and whether such punishment still serves retributive and deterrence purposes.

The Court remanded Madison’s case to the lower courts for additional proceedings to determine whether he was incompetent for execution in light of the Court’s clarification of the role dementia plays in CFE determination. However, Madison passed away in prison in 2020 before he could be evaluated under the Court’s refined CFE standard. Given the aging death row population, we expect additional cases of incompetency due to dementia will arise.

### 1.2. Empirical Legal Studies

Having briefly reviewed the historical development of the concept of CFE, particularly how its interpretation and application have been shaped by the English common law tradition and subsequent Supreme Court cases, we now turn to empirical studies on CFE, an area that remains significantly underexplored.

Blume et al. (2014) conducted the first systematic empirical study on CFE, analyzing all *Ford* claims decided by courts from 1986 to mid-2013. Their findings unveiled the black box of CFE and revealed several counterintuitive insights. Notably, as Table 1 shows, they found that, as of their publication, only 141 death row inmates (2% of those sentenced to death between 1986 and 2012) filed *Ford* claims despite 1280 executions during this period. Furthermore, only 21 of these claims were successful, indicating that the individuals were deemed incompetent. This underscores the underutilization of *Ford* claims, even among those eligible, as well as the alarmingly low success rate of such claims. In a similar vein, they found no notable differences in the filing rate of *Ford* claims before versus after the *Panetti* ruling. The rate remained roughly the same, with 8.4% six years before *Panetti* (2001–2007) and 7.6% six years after (2007–2013) (Blume et al., 2014, Table 3). Thus, contrary to some expectations, there was no significant increase in filings following *Panetti*, as the overall filing rate remained consistently low, creating “a dearth of frivolous claims”.

They then investigated the mental health history of the claimants and found that in the cases where courts reached the merits of the CFE claim (92 cases), 62% of the claimants had a documented history of delusions, schizophrenia, or both, while 18% of the cases were suspected of malingering. Additionally, an average of 59.8% of the cases had prior challenges regarding non-CFE competency, mostly related to competency to stand trial. Among the successful *Ford* claimants, 76.2% had filed previous non-CFE competency challenges, and 23.8% were found incompetent at an earlier point in the proceedings in those cases. In contrast, 54.9% of unsuccessful CFE claimants had filed such challenges in prior litigation, and 11.3% were found incompetent in those previous claims (Blume et al., 2014, Table 5).

Next, they revealed notable disparities in *Ford* claim outcomes across states. This study highlighted wide-ranging incompetency findings, varying from 0% to 21.9% to 100% among different states (Blume et al., 2014, Table 4), revealing concerning variances in terms of the CFE evaluation between states. Likewise, disparities were found in terms of the race of the claimants (Blume et al., 2014, Table 6). While the filing rate generally reflected the racial composition of the death row population, showing no drastic differences, the success rate revealed that African American claimants were more likely to be deemed incompetent (31.6%) compared with White claimants (11.6%). The driving mechanisms behind this difference remain unclear, though they may be influenced by the larger proportion of White *Ford* claimants or by other racial disparities in treatment and evaluations.

Blume et al. (2014) underscore the potential influences of demographic, sociopolitical, geographic, and litigation factors on CFE decisions, highlighting the complexities involved in CFE evaluations. The authors also question the number of incompetent CFE claimants, expressing concern that the current “porous” CFE standards inadequately protect mentally ill inmates and advocate for more rigorous CFE evaluations.

Using a similar approach but from a different perspective, Perlin, Harmon, and their colleagues published a trilogy of empirical papers on CFE over the past four years. Perlin and Harmon (2021) initially focused on CFE cases within the Fifth Circuit and related district courts, as the Fifth Circuit has been widely criticized for disregarding the Supreme Court’s decision in similar past decisions, such as *Atkins v. Virginia* (2002) on cases regarding banning executing individuals with intellectual disabilities. Their study examined 13 cases in total, including nine Fifth Circuit cases and four district court cases, and revealed that in the 14 years since the *Panetti* ruling, no cases in the Fifth Circuit have resulted in a finding of incompetence for execution. Only two “victories” have occurred at the district court level. According to the authors, the unsuccessful CFE cases within the Fifth Circuit were rejected for four primary reasons: (1) the battle of experts, which included issues regarding the credibility of expert witnesses (*n* = 5) and the inadequacy of expert funding (*n* = 3); (2) malingering (*n* = 3), which included issues of suspecting the claimant of feigning symptoms; (3) involuntary restorative treatment (*n* = 3), which related to synthetic competency; and (4) insufficient presentation of mental illness evidence (*n* = 2). The findings underscored the Fifth Circuit’s perceived lack of empathy—characterized as having “no soul”—for its failure to deem even a single death row inmate incompetent for execution despite compelling evidence of mental illness.

In their second paper of the trilogy, Perlin et al. (2022) expanded their review to include all available federal CFE cases, enabling them to compare the Fifth Circuit’s rationale to that of other circuits. In their study, they identified 14 non-Fifth Circuit CFE cases. Among these, only one, which they referred to as an “evanescent victory,” resulted in a finding of incompetency. The case they referred to was *Madison v. Commissioner* (2017), a previous case filed by Vernon Madison in federal court that resulted in a finding of incompetency, which was later vacated on procedural grounds. Madison refiled his CFE claim in the Alabama state courts, and as discussed above, the Supreme Court issued landmark *Madison v. Alabama* (2019). When examining the majority of the unsuccessful non-Fifth Circuit CFE cases, the reasons for rejecting the *Panetti* claims were as follows and were similar to the findings in their 2021 paper: 50% of claims involved issues related to the credibility of the experts, 14% were based on concessions made by the defense experts, 14% acknowledged mental illness but found no evidence of a lack of rational understanding linking the crime and execution, and 7% were attributed to malingering. Combined with their findings on cases in the Fifth Circuit, and further dismayed by the harsh decisions regarding CFE in federal courts, Perlin et al. concluded that *Panetti* “has had absolutely no impact on defendants in federal habeas corpus proceedings” and that the “Supreme Court decisions have turned out to be illusory”.

Most recently, Perlin et al. (2023) completed their trilogy and expanded their scope by examining CFE cases in state courts. Their primary analysis included 29 case opinions involving 24 death row inmates who filed CFE claims. Within the cohort of 24 death row inmates with Panetti claims, five were successful. Among the rejected cases, the main reasons for rejection were “concessions by defense experts” (*n* = 7), “defense witness credibility” (*n* = 7), “malingering,” and “mentally ill but rational,” reflecting findings from the previous two papers. They concluded that “the continued reliance on cognitive-simplifying heuristics and false ordinary common sense—along with a failure to recognize the significance of therapeutic jurisprudence principles—taints the entire area of law,” and that the pattern in the Fifth Circuit and other federal circuits “has been replicated in the state courts”.

### 1.3. The Present Study

By building on existing empirical CFE studies, several trends emerge. First, there is a small number of death row inmates who file *Ford* claims, with outcomes potentially influenced by sociopolitical factors, as seen in variations across states and racial groups (Blume et al., 2014). Second, the success rate is low—and some would argue alarmingly so (Blume et al., 2014; Perlin & Harmon, 2021; Perlin et al., 2022, 2023)—with common reasons for rejection including expert witness credibility, malingering, and the stringent determination of “mentally ill but rational” (Perlin & Harmon, 2021; Perlin et al., 2022, 2023)—it is evident that empirical research on CFE remains in its early stages.

Despite its significance as a life-and-death decision, the multi-facets of CFE remain underexplored; specifically, a critical intellectual gap remains: the absence of a comprehensive analysis of the characteristics of both the individuals and the crimes involved in successful *Ford* claims. A close review of successful *Ford* claimants and their capital cases is necessary. While existing studies provide a broad perspective on historically successful *Ford* claims, little is known about the commonalities among these cases and the claimants’ experiences within the criminal legal system. Specifically, identifying shared characteristics among successful *Ford* claimants—a unique population that has “survived” the *Ford* claims process within a largely unyielding “soul-less” criminal justice system—could provide valuable insights into the utilization of CFE and the factors influencing execution exemptions.

Drawing inspiration from empirical legal studies on small-sample death row populations (e.g., women on death row in the United States, *n* = 48; Babcock et al., 2024), this study aimed to be the first comprehensive analysis of individuals sentenced to death who have filed and won *Ford* claims. Following Babcock et al. (2024), we sought to conduct a “holistic and intersectional” examination of the factors influencing the execution and non-execution of individuals with serious mental illnesses on death row. Specifically, we aimed to examine three key facets of successful *Ford* claimants and their cases: (1) the characteristics of successful *Ford* claimants, including demographics, mental illness diagnoses, and mental health conditions; (2) their crimes of conviction, such as offense year, jurisdiction, victim demographics, and offender-victim relationship; and (3) the professional considerations regarding successful *Ford* claims, including mental health evidence presentation, discussions on malingering (Blume et al., 2014), prior adjudicative competence evaluations (Blume et al., 2014), and the history of *Ford* claims and evaluators. Although this study was necessarily a small-scale descriptive analysis due to its limited sample size, it represents the largest sample of successful *Ford* claimants to date and provides a foundation for future researchers to develop hypotheses as more data become available. Note that for some aspects of our analysis, we provided comparisons between our target group and other similar groups (e.g., the general death row population, the male incarcerated population, general homicides, and, when data are available, the nationwide general population), especially in the context of sentencing and the death penalty, to help readers understand this unique group.

## 2. Method

### 2.1. Data Collection

Our primary dataset consists of publicly available case files, primarily state, appellate, and federal court decisions, for successful *Ford* claimants–individuals who meet our inclusion criteria: (1) they have been sentenced to death; (2) they have filed a *Ford* claim in which their CFE was evaluated; (3) they were deemed incompetent for execution (“succeeded in their *Ford* claim”) in at least one CFE evaluation, as some cases involve multiple evaluations; (4) their successful *Ford* claims occurred in any jurisdiction or court level; and (5) their cases span the entire history of the United States.

We identified successful *Ford* claimants through a multi-stage process. First, in mid-2019, we compiled a list of 21 successful *Ford* claimants from Blume et al.’s (2014) database. Next, with input from the second author—a law school professor and a capital defense attorney—we consulted a network of scholars, attorneys, and non-governmental and non-profit organizations specializing in capital cases nationwide. These experts provided names of individuals known to have filed and won *Ford* claims, leading us to identify an additional seven successful claimants.^1^ Then, we reviewed Perlin and colleagues’ trilogy of papers (Perlin & Harmon, 2021; Perlin et al., 2022, 2023) to determine whether any new successful *Ford* claims should be included. However, we did not identify any additional cases, as all the successful cases mentioned in their papers had already been identified in our previous round of review. After compiling the list of 28 individuals, we conducted a comprehensive search in Westlaw Edge and Lexis Advance for cases citing *Ford v. Wainwright* (1986), systematically reviewing them to confirm that each case met our inclusion criteria—specifically, that the individual was deemed incompetent for execution after filing a *Ford* claim. This search did not yield any additional successful cases. By mid-2024, at the time of analysis, we had identified a total of 28 successful *Ford* claimants, which, to the best of our knowledge, represent the entire population of our target group.

After identifying the names of successful *Ford* claimants, the research team retrieved all available case files from Westlaw Edge and Lexis Advance, which served as the primary sources of information for these cases. To supplement demographic data, we also consulted the Death Penalty Information Center’s Death Penalty Census Database (https://deathpenaltyinfo.org/facts-and-research/data/death-penalty-census, accessed on 15 December 2023) and relevant news coverage.

### 2.2. Data Coding

We developed a coding scheme—a list of variables of interest—based on Blume et al. (2014)’s original framework, which we revised to align with our research questions. Each case was initially coded by at least one research assistant, with a second research assistant reviewing the coding for accuracy. Any discrepancies, which were rare, were resolved through group discussions to ensure consistency and reliability.

### 2.3. Data Validation

After coding, two senior research assistants (H.T.D. and N.M.B.) converted the case and claimant information into narrative summaries, which were then shared with the defense teams of the claimants, including attorneys and paralegals, for accuracy verification. This validation process took place between late 2022 and mid-2024. Most teams responded promptly, allowing for revisions based on their feedback. We employed this double-checking method to ensure data accuracy while maintaining client-attorney confidentiality. Although we do not have access to the full legal files and medical histories of the claimants, we gathered relevant information and consulted professionals familiar with these cases to verify the coding of variables, thereby addressing potential gaps caused by missing data.

### 2.4. Data Analysis

Data organization, presentation, and descriptive analyses were conducted using Microsoft Excel 365, which has built-in functions and formulas to calculate summary statistics and create charts and graphs. Figure generation was performed using R (version 4.2.2).

### 2.5. Ethics

We obtained exemption approval from the Institutional Review Board at Cornell University for the data verification process. In accordance with requests from defense attorneys involved in these cases, we withheld the names of individual claimants and provided only aggregate results, omitting specific identifiers due to ongoing *Ford* claims or related disputes.

## 3. Results

Although this is a small-scale descriptive study, it comprises the largest sample of successful *Ford* claimants to date and serves as a source of hypotheses for future researchers to pursue as more data become available. Thus, we offer the following results as a “think piece” that is not as definitive a claim but rather a basis for researchers to pursue this topic in greater depth and quantitative sophistication as more data become available.

### 3.1. Characteristics of Successful Ford Claimants

#### 3.1.1. Demographics

**Gender.** All 28 successful *Ford* claimants are male; this pattern mirrors the gender distribution of the death row population, where 97.9% of inmates are male (Bureau of Justice Statistics, 2023). It is worth noting, however, that there have been female *Ford* claimants. Unfortunately, all their claims were unsuccessful, resulting in no female individuals being recognized incompetent for execution to date. In view of the demographics of death row inmates, we do not expect the gender composition of claimants to change greatly with the availability of more data.

**Figure 1:**
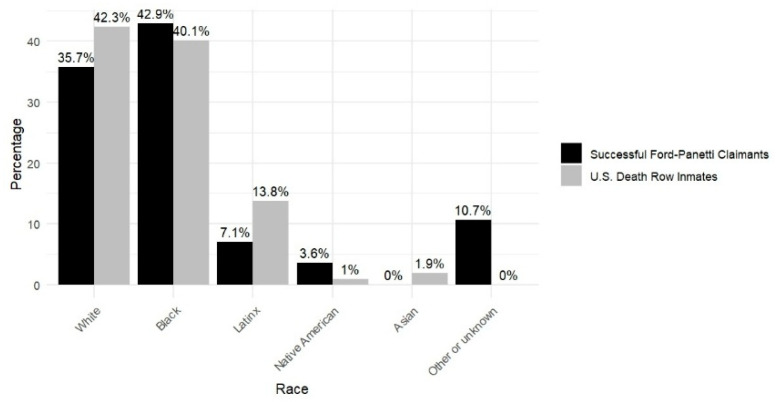
Successful *Ford* claimants vs. U.S. death row inmates by race.

**Race.** The racial composition of the successful *Ford* claimants is as follows: White (*n* = 10; 35.71%), Black (*n* = 12; 42.86%), Latinx (*n* = 2; 7.41%), Native American (*n* = 1; 3.57%), Mixed Race (*n* = 1; 3.57%), Other (*n* = 1; 3.57%), and Unknown (*n* = 1, 3.57%). Due to the small number of cases in each racial category, these percentages could change with additional data. In terms of race, as shown in Figure 1, the racial composition of successful *Ford* claimants differs from that of the general death row population (National Association for the Advancement of Colored People, 2022). Specifically, the proportion of successful Black *Ford* claimants (42.9%) closely mirrors that of the general Black death row population (41.0%). Native American claimants (3.6%) are overrepresented compared with the general Native American death row population (1.0%). Conversely, White (35.7%) and Latinx (7.1%) successful *Ford* claimants are underrepresented relative to the general White (42.3%) and Latinx (13.8%) death row populations. This finding aligns with Blume et al. (2014), who reported that Black *Ford* claimants constitute a notably larger proportion of those deemed incompetent for execution compared with their White and Latinx counterparts. Similarly, the present study identified a pattern of underrepresentation among White and Latinx claimants. If this could be replicated with a larger data corpus, it would represent an unexpected example of pro-Black incarceration outcomes.

**Figure 2:**
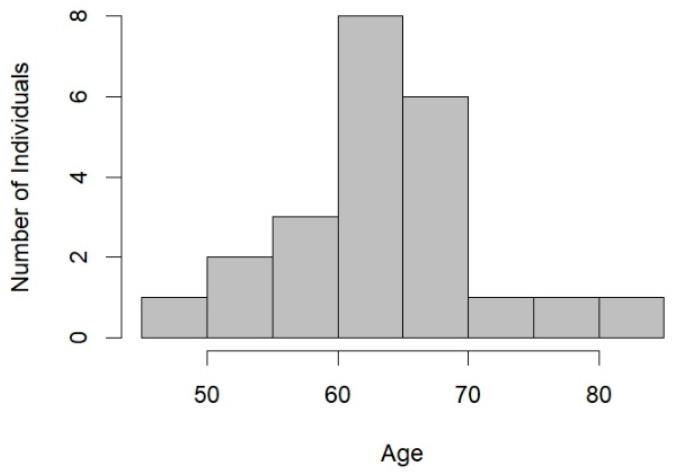
Successful *Ford* claimants by age.

**Figure 3:**
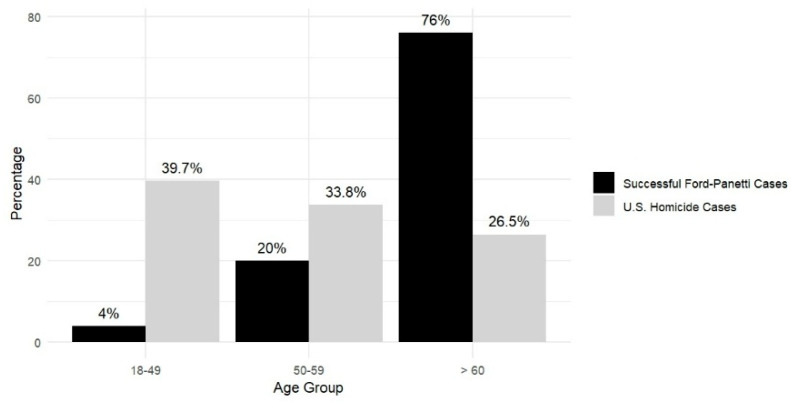
Successful *Ford* claimants vs. U.S. death row inmates by age.

**Age, Time on Death Row, and Current Status.** Excluding the three successful *Ford* claimants who died for causes unrelated to execution, the average age of the 25 living successful *Ford* claimants, with two missing data, is 64.7 years (*n* = 23) at the time of analysis. As shown in Figure 2, among them, one claimant is under 50 years old, five claimants are between 50 and 59, 13 are between 60 and 69, and four are older than 70. The median age is 65 years, with a range from 49 to 82 years. The average duration of their time on death row is 34 years, ranging from 12 to 47 years. (For the three claimants who died on death row, the average duration of their time on death row was 32 years, with a range from 31 to 33 years.) As shown in Figure 3, the successful *Ford* claimants are relatively older than the typical death row inmate, with a mean age of nearly 65. Of these, 96% are 50 or older, and 76% are 60 or older. In contrast, national data indicate that the average age of death row inmates is 51, with 60.3% aged 50 or older and 26.5% aged 60 or older (Bureau of Justice Statistics, 2023).

**Figure 4:**
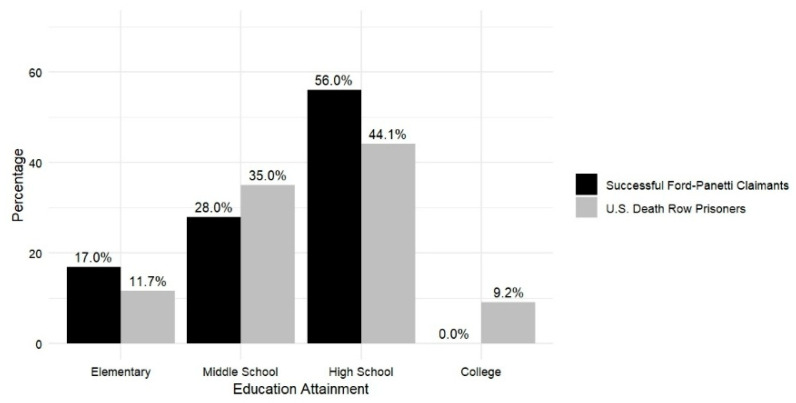
Successful *Ford* claimants vs. U.S. death row inmates by education.

**Education.** The educational attainment of successful *Ford* claimants is as follows (*n* = 18; 10 missing data): None have completed any college education. The majority (*n* = 10; 56%) have attained a high school diploma, while 28% (*n* = 5) have reached the middle school level. Notably, 17% (*n* = 3) have only completed elementary education. Due to the temporal changes in educational attainment, we anticipate that future claimants will have more years of formal education than past claimants, who mostly completed their schooling by the mid-twentieth century. As claimants possess greater educational attainment, it will be interesting to see if this affects the success rate. As shown in Figure 4, successful *Ford* claimants exhibit lower levels of educational attainment compared with the broader death row population. Although the high proportion of missing data limits definitive conclusions, the available evidence, slender as it is, reveals notable disparities: none of the successful *Ford* claimants have completed a college degree, and a higher proportion have only completed elementary education. In contrast, among the general death row population in the United States, 9.2% have completed a college degree, and 11.7% have completed only an elementary education, with a median education level of 12th grade (Bureau of Justice Statistics, 2023). As we noted, this is a trend that we anticipate may change with time, as the mean educational attainment of Americans has been steadily increasing.

**Employment.** The employment status of successful *Ford* claimants is as follows (*n* = 19; 9 missing data): The majority were employed full-time or part-time (*n* = 14) at the time of the offense, while a smaller number were not employed (*n* = 2). Additionally, some individuals were in other situations, such as custody or incarceration (*n* = 3), as the crimes they committed occurred while they were incarcerated. Although national data on the employment records of death row inmates are unavailable for baseline comparison, this finding aligns with previous research suggesting that while employment is often considered a protective factor against crime, it does not necessarily prevent the commission of severe offenses (Lee, 2019).

**Prior Criminal Record.** The prior criminal record of successful *Ford* claimants is as follows (*n* = 19; 9 missing data): the majority had previous criminal convictions (*n* = 13), while 6 had no prior criminal history. This pattern mimics the broader death row population, where over half of inmates have prior criminal records (Bureau of Justice Statistics, 2023). Although the numbers are too small to be confident, going forward, this will be an area that can be updated as new cases come online.

#### 3.1.2. Mental Illness Diagnoses and Mental Health Conditions

All 28 (100%) successful *Ford* claimants were found to have been diagnosed with at least one serious or severe mental illness (SMI),^2^ which is markedly higher than the 10–16.7% of those in prison or jail (Bureau of Justice Statistics, 2006; Steadman et al., 2009) and 6.0% in the general population (National Institute of Mental Health, 2024).

Among SMIs, we focused specifically on schizophrenia,^3^ substance use disorder (SUD),^4^ and personality disorder (PD),^5^ including antisocial personality disorder, as these SMIs have a long-standing history of involvement in the context of sentencing decisions and the death penalty (French, 1994; Sevilla, 1999; Slobogin, 2000), and many of our interested groups were diagnosed with these psychoses (PD, at least in our sample, always co-existed with other psychoses). Given their long-standing legal and ethical implications, understanding the prevalence of these SMIs among successful *Ford* claimants provides critical insight into the intersection of mental illness and CFE. Once again, we reiterate the caveat we have raised previously: the small number of cases precludes multivariate analyses needed to forge policy recommendations, a limitation we hope future researchers may be able to surmount.

**Schizophrenia.** Among the successful *Ford* claimants with available schizophrenia-related information (*n* = 25; 3 missing data), 24 claimants were diagnosed with schizophrenia based on the diagnostic criteria at the time of their evaluation, while one claimant was not. As Table 2 shows, there is an alarmingly high rate of schizophrenia among the successful *Ford* claimants (86%) compared with the United States state prison population (2% to 6.5%; Prins, 2014) and the general population’s 12-month prevalence (0.33%) and lifetime prevalence (0.48%) (Simeone et al., 2015).

**Substance Use Disorders.** Among the successful *Ford* claimants with available SUD information (*n* = 22; 6 missing data), eight claimants were diagnosed with SUD based on the diagnostic criteria at the time, while the remaining 14 claimants did not receive such a clinical diagnosis. Of the non-SUD claimants, at least one was known to be drinking heavily, though they did not meet the criteria for a clinical diagnosis. As Table 2 shows, the prevalence of SUD among the successful *Ford* claimants mirrors the prevalence among general male prisoners (30%; Fazel et al., 2017) but is much higher than the general population’s 1-year prevalence (6.6%) and lifetime prevalence (13.2%) (Somers et al., 2004).

**Personality Disorders.** Among the successful *Ford* claimants (*n* = 23; 5 missing data), 11 claimants were diagnosed with personality disorders, including at least three cases of antisocial personality disorder, while the remaining 12 had no such diagnoses. As Table 2 shows, there is a high rate of PD among successful *Ford* claimants (39%) compared with the 21% prevalence in the sentenced male population (Rotter et al., 2002) and 9.1% in the general population (Lenzenweger et al., 2007).

**Other SMIs.** In addition to schizophrenia, SUD, and PD, among the successful *Ford* claimants with available data (*n* = 25; 3 missing data), 20 were diagnosed with SMIs outside the three previously mentioned SMI categories, while five were not, including depressive disorders (*n* = 8), neurocognitive disorders^6^ (*n* = 6), bipolar and related disorders (*n* = 2), anxiety disorders (*n* = 1), neurodevelopmental disorders (*n* = 1), and obsessive-compulsive and related disorders (*n* = 1). Among these SMIs, specifically, as Table 2 shows, the prevalence of depressive disorders (29%) mirrors that of the prison population (36.9%) (Bedaso et al., 2020) but is much higher than the general population’s prevalence (8.3%) (National Institute of Mental Health, 2024).

**Psychiatric Comorbidity.** Of the 28 successful claimants, 22 were diagnosed with more than one SMI. Table 3 presents the psychiatric comorbidity patterns among these claimants: while most have schizophrenia, they are often also diagnosed with one or more SMIs, such as SUD, PD, and others; the prevalence of psychiatric comorbidity is higher than the lifetime prevalence in the general population, where 27.7% have two comorbid conditions, and 17.3% have three (Al-Asadi et al., 2015).

**Intellectual Disability.^7^** In addition to SMIs, our attention now shifted to another central issue in the context of mental health in capital cases: intellectual disability (Ceci et al., 2003; Hritz et al., 2019). The imposition of the death penalty on individuals with intellectual disabilities has sparked significant debate, particularly in relation to the Eighth Amendment. Following the Supreme Court’s decision in *Atkins v. Virginia* (2002), which prohibited the execution of individuals with intellectual disabilities, the categorical exemption has made the evaluation of intellectual disability a critical factor in determining eligibility for the death penalty. However, in the present study, many cases were sentenced prior to the landmark *Atkins* decision, which makes the discussion of intellectual disability still highly relevant. Among the successful *Ford* claimants with available intellectual disability information (*n* = 22; six cases with missing data), six claimants were diagnosed with intellectual disability (previously referred to as mental retardation), while 15 claimants were not. In one case, the attorney initially presented evidence of the defendant’s intellectual disability but later withdrew it. As Table 2 shows, the prevalence of intellectual disability among successful *Ford* claimants (21%) is higher compared with a 10% prevalence in young male prisoners (Herrington, 2009) and 1.65% in the general population (Centers for Disease Control and Prevention, 2023).

**Onset of Mental Illness.** After providing an overview of the mental health diagnoses of the successful *Ford* claimants, we now turn to the timing of the onset of their mental illness. Specifically, we examine whether the illness began before the offense or developed later, such as during incarceration. Among the successful *Ford* claimants with available onset timing data (*n* = 25; three cases with missing data), 22 claimants exhibited signs of mental illness before committing their offense, while three showed signs only after the offense, as 79% of the claimants presented evidence of SMIs at the time of their offense. In comparison, 11% presented evidence of SMIs for the first time after the offense and after they were sentenced to death. We argue that this is likely true for two reasons: first, some SMIs are not detected at trial due to various reasons, including potential instances of ineffective counsel; the second cause is due to prolonged incarceration under harsh circumstances—classic examples of death row syndrome. We believe this contrasts with many death row cases, where individuals are typically not mentally ill at the outset but may develop mental health issues over time due to prolonged incarceration.

**Treatment.** After providing an overview of the mental health diagnoses, onset time, and malingering of the successful *Ford* claimants, we now turn to their treatment. Among the 28 successful claimants with available treatment information (*n* = 22; 6 cases with missing data), 16 claimants received some form of treatment for their mental illness either before the offense or while incarcerated, while six claimants either did not undergo treatment or refused it, only about half of the successful *Ford* claimants (57%) have ever received any form of treatment, which is slightly lower than the treatment rate for the general SMI population in the United States (66.7%) (National Institute of Mental Health, 2024). This discrepancy may be attributed to the inadequate mental health resources in prisons, as well as the controversy surrounding restored competency, as many claimants and their defense teams may have strategically refused treatment due to concerns about facing the implications of improved mental status.

### 3.2. Successful Ford Claimants’ Crime of Conviction

#### 3.2.1. Offense Year

**Figure 5:**
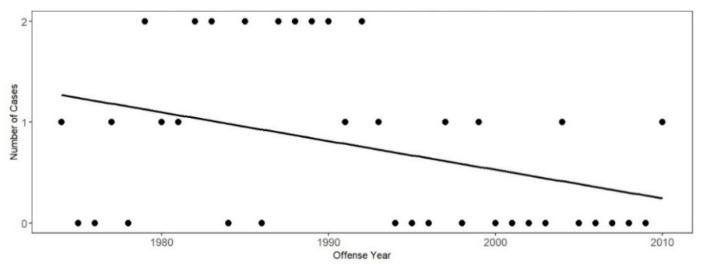
Successful *Ford* cases by offense year with trajectory analysis.

The number of years of conviction for crimes of successful *Ford* claimants spans from 1974 to 2010. Figure 5 shows that the number of successful *Ford* cases fluctuates, ranging from zero to two cases per year, with a declining trend via trajectory analysis. Of course, there is the possibility that temporal trends may be altered in the future as more data become available, so this apparent past trend may not continue.

#### 3.2.2. State

Next, we examined the jurisdictional state of these successful *Ford* cases, as presented in Table 4. Out of the 27 states that have retained the death penalty (including five states that still have the death penalty but have paused executions through executive action), 13 have deemed at least one *Ford* claimant incompetent for execution. Texas has the highest number of successful *Ford* cases, totaling nine, followed by Oklahoma with three. Arkansas, Idaho, Louisiana, Pennsylvania, and South Carolina each have two cases. Additionally, Arizona, California, Missouri, Mississippi, North Carolina, and Ohio each have one successful *Ford* case. The remaining 14 states that retain the death penalty and do not have recorded cases of individuals deemed incompetent for execution are Alabama, Florida, Georgia, Indiana, Kansas, Kentucky, Montana, Nebraska, Nevada, Oregon, South Dakota, Tennessee, Utah, and Wyoming. Therefore, around half—13 out of 27, or approximately 48%—of the states that retain the death penalty have found at least one person to be incompetent for execution, while just over half—14 out of 27, or about 52%—have never done so. It is important to note that, as we are only presenting the outcomes of successful *Ford* claims, there may be cases that were ruled on but ultimately denied. Finally, the sheer limited amount of data renders the conclusion tentative that roughly half of the states with the death penalty have ruled CFE at least once. Going forward, this temporal trend is an important hypothesis that could be pursued if additional data become available.

To put into context the above finding, we examined the ratio of the number of successful *Ford* claims in comparison to the number of prisoners on death row and the number of executions in states that have ruled on successful *Ford* claims. Table 5 shows notable variations across states in terms of the ratio of the number of successful *Ford* cases to the number of death row inmates: Arizona reported one case, constituting 0.9% of its total death row population. Arkansas had two cases, accounting for 7.1%, while California reported one case, representing 0.2%. Idaho had the highest percentage, with two cases, amounting to 25.0% of its death row inmates. Louisiana and Mississippi each reported 2 cases, representing 3.2% and 2.8%, respectively. Missouri reported one case, accounting for 5.6%, and North Carolina and Ohio reported one case each, constituting 0.7% and 0.8%, respectively. Oklahoma reported three cases, amounting to 7.5%; Pennsylvania reported two cases, representing 1.6%; and South Carolina reported two cases, accounting for 5.6%. Texas had the highest number of cases, with nine cases constituting 4.7% of its death row population. Thus, on average, across the states examined, approximately 5% of death row inmates were deemed incompetent for execution in states that have ruled on successful *Ford* claims. However, the variation among states in the percentage of successful *Ford* claims is substantial, ranging from a high of 25.0% in Idaho to a low of 0.2% in California, so this could be profitably revisited as more *Ford* claims are decided.

In the same vein, Table 6 also shows notable variations across states where persons on death row have been found incompetent to be executed in terms of the ratio of the number of successful *Ford* cases to the number of executions: Idaho and Pennsylvania stand out with the highest percentages of incompetent for execution cases at 66.7%, despite having conducted only three executions each since 1976. Conversely, Missouri and Texas show relatively lower percentages of incompetence cases at 1.0% and 1.5%, respectively, while reporting higher numbers of executions, totaling 99 and 587, respectively. Other states, such as Arkansas, California, Louisiana, Mississippi, North Carolina, Ohio, Oklahoma, and South Carolina, display varying percentages of incompetence cases ranging from 1.8% to 7.7%, with execution counts ranging from 13 to 124. On average, across the states examined, approximately 13.5% of those who faced executions have secured a *Ford* claim. Hence, we observed a variation among states in terms of the ratio of successful *Ford* cases to death row inmates and executions.

#### 3.2.3. Victim Demographics

**Figure 6:**
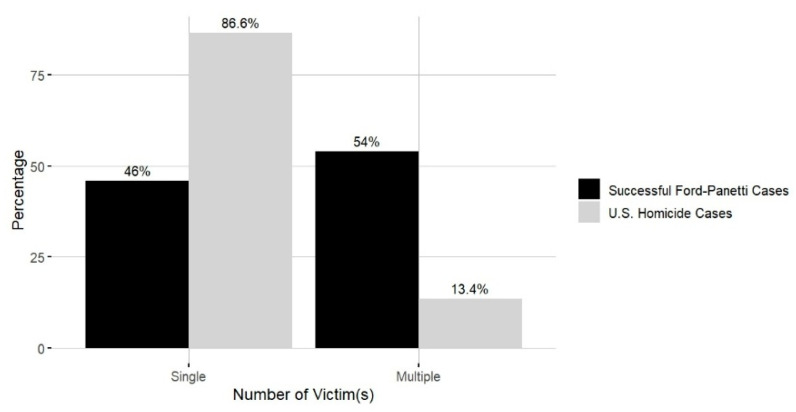
Victims of successful *Ford* cases vs. U.S. homicide cases by number.

**Number of Victims.** The victim counts across the 28 successful *Ford* cases revealed that 13 cases involved a single victim (46%), while 15 cases involved multiple victims (54%), resulting in a total of 60 victims. The distribution of victim cases was as follows: one victim (*n* = 13; 46%), two victims (*n* = 11; 39%), three victims (*n* = 2; 7%), five victims (*n* = 1; 4%), and 14 victims (*n* = 1; 4%). (One of the cases involving two victims included an unborn child.) Out of the 60 victims, 56 were deceased, while four remained alive after the offense. As in Figure 6, the distribution differs from the national homicide trend (86.6% single-victim cases vs. 13.4% multiple-victim cases, Federal Bureau of Investigation, 2024), highlighting the higher proportion of multiple-victim cases in successful *Ford* cases. Notably, none of the multiple-victim cases involved indiscriminate violence, such as random mass murders or serial killings. The most concerning case, involving 14 victims and reflecting the pattern of multiple-victim cases in successful *Ford* cases, was primarily directed at the claimant’s family members, including his own children, rather than being a random or indiscriminate attack.

**Figure 7:**
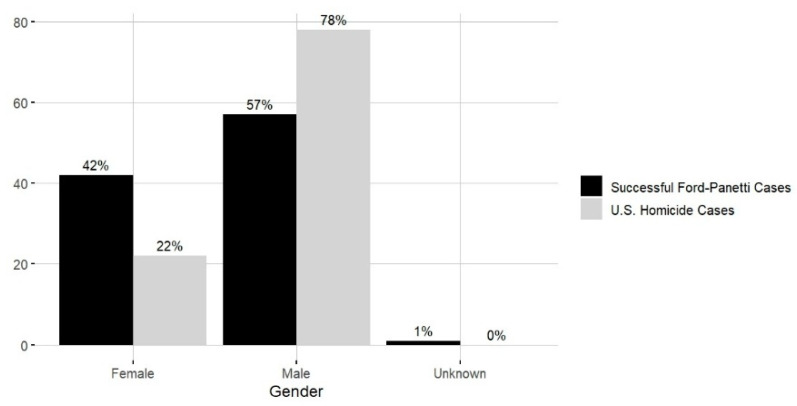
Victims of successful *Ford* cases vs. U.S. homicide cases by gender.

**Victim Gender.** The gender distribution of victims in successful *Ford* cases (*n* = 59, with one missing data) was as follows: A total of 42% (*n* = 25) were female, and 57% (*n* = 34) were male. Fourteen cases involved only male victims, and five involved only female victims. Nine cases involved victims of mixed genders or where the gender was unknown. The gender distribution of victims differs between successful *Ford* cases and national homicide data (Figure 7), indicating a higher proportion of female victims among successful *Ford* cases.^8^

**Figure 8:**
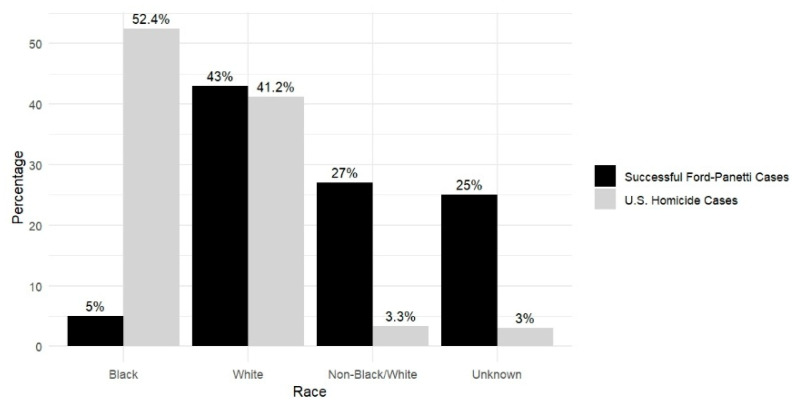
Victims of successful *Ford* cases vs. U.S. homicide cases by race.

**Victim Race.** The racial breakdown of the victims in successful *Ford* cases was as follows: 5% Black (*n* = 3), 43% White (*n* = 26), 12% Latinx (*n* = 7), 3% Native American (*n* = 2), 12% Other (including Mixed Race) (*n* = 7), and 25% Unknown (*n* = 15). Figure 8 reveals differences between the successful *Ford* cases and national homicide data (Federal Bureau of Investigation, 2024), characterized by a lower proportion of Black victims and a higher proportion of Non-Black/White victims and victims of unknown race.^9^ Collapsing across the data from the 24 available claimants’ race and victims’ race, seven of these 24 cases involved intraracial murder (for example, four cases with White defendants and White victims), and 17 cases involved transracial murder (such as eight cases with Black perpetrators and White victims; one case involved White perpetrators with Black victims). One case involved both interracial and transracial murders. This is an area in which important intersectional analyses will depend on much larger samples.

**Figure 9:**
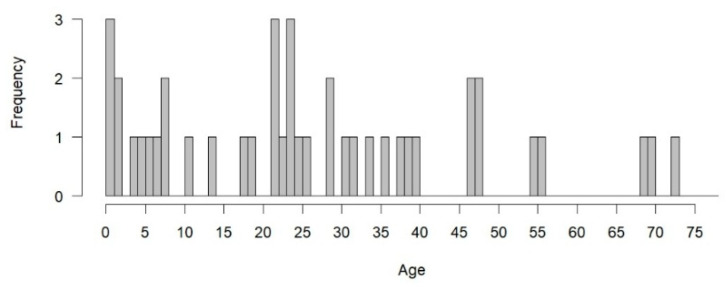
Victims in successful *Ford* cases by age at the time of the offense.

**Figure 10:**
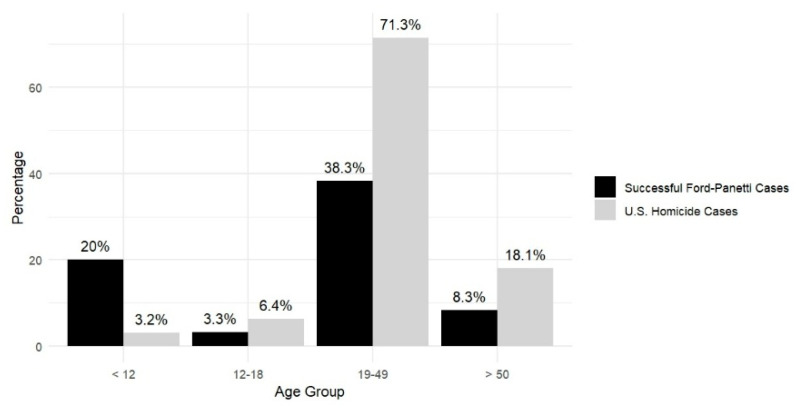
Victims of successful *Ford* cases vs. U.S. homicide cases by age.

**Victim Age.** For victims in successful *Ford* cases with available age data (Figure 9), the mean age at the time of the offense was 25.8 years (*SD* = 19.1), including one case where the age was recorded as 0 for an unborn child. Figure 10 illustrates the age distribution of the victims: 20% (*n* = 12) were under the age of 12 (including the unborn child), 3.3% (*n* = 2) were between 12 and 18 years old, 38.3% (*n* = 23) were between 19 and 49 years old, 8.3% (*n* = 5) were over the age of 50, and 30% (*n* = 18) had an unknown age. After excluding cases with unknown data, as shown in Figure 10, the victims of successful *Ford* claimants include a higher proportion of child victims under the age of 12, half of whom are the claimants’ family members, and a substantially lower proportion of adult victims over the age of 18 than national data (Federal Bureau of Investigation, 2024).

#### 3.2.4. Claimant–Victim Relationship

**Figure 11:**
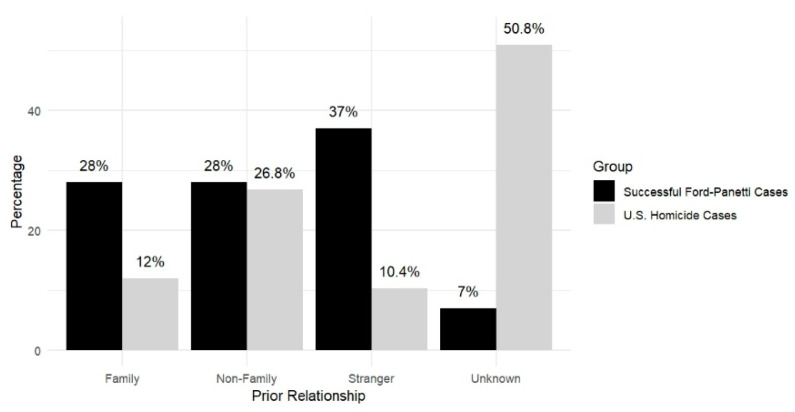
Offender–victim relationship by prior relationship in successful *Ford* cases vs. U.S. homicide cases.

The claimant–victim relationships in the successful *Ford* cases are as follows: 28% (*n* = 17) of the victims were family members (e.g., spouses, parents, children, siblings, relatives, in-laws); 28% (*n* = 17) were friends or acquaintances of the claimants; 37% of the victims (*n* = 22) were strangers to the claimants; and 7% (*n* = 4) were unknown. As shown in Figure 11, when identifying a difference from the national data (excluding cases with unknown data) (Federal Bureau of Investigation, 2024), a higher proportion of victims were killed by family members or strangers in the successful *Ford* cases. Additionally, we found that contrary to national trends (Federal Bureau of Investigation, 2019), intraracial homicide—typically the majority of homicide cases nationwide—was less prevalent among the successful *Ford* cases. As Table 7 shows, while national data indicate that same-race homicides account for 79% of White victim deaths (White-on-White homicides) and 89% of Black victim deaths (Black-on-Black homicides), in the present study, the pattern was almost reversed, with 32% of cases involving intraracial offenses and 77% of cases involving transracial offenses. We further examined the claimant–victim relationship by considering their prior relationship and the gender of the victims. Compared with the national data (excluding cases with unknown data) (Bureau of Justice Statistics, 2021), as shown in Table 8, the findings reveal different patterns between the present study and the national trend. In the present study, the proportion of female victims murdered by family members is much lower than in the national sample, while the proportions murdered by non-family-non-strangers and strangers are higher. Conversely, the proportion of male victims murdered by family members or strangers is higher than in the national sample, whereas the proportion of those murdered by non-family-non-strangers is lower.

### 3.3. Professional Considerations of Successful Ford Claims

#### 3.3.1. Mental Health Evidence Presentation

**Figure 12:**
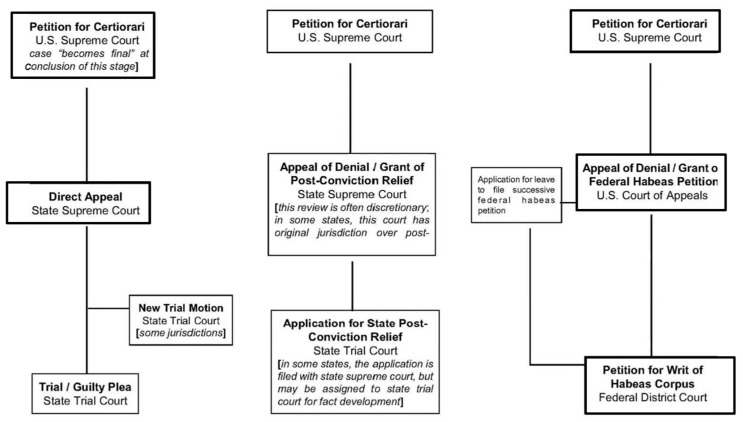
Three phases of the review of capital cases in the United States.

We examined whether mental health evidence of the successful *Ford* claimants was presented at various phases of legal proceedings:^10^ the trial phase, the post-conviction relief (PCR) phase, and the habeas corpus phase (Figure 12; Blume, 2021). We found that, as Table 9 presents, during the trial phase, among successful *Ford* cases with available mental health evidence data (*n* = 24, with four cases missing data), mental health evidence was presented in 17 cases and absent in seven cases. During the PCR phase, for successful *Ford* cases with available mental health evidence data (*n* = 19, with nine cases missing data), mental health evidence was presented in 18 out of 19 cases. During the habeas corpus phase, among successful *Ford* cases with available mental health evidence data (*n* = 26, with two cases missing data), mental health evidence was presented during the habeas corpus phase in all 26 cases.

#### 3.3.2. Discussion on Malingering

Next, we examined the issue of malingering in successful *Ford* claims, as malingering is traditionally a central concern in cases involving mental health evidence. In the present study, among the successful *Ford* claims with available malingering data (*n* = 21, with seven cases missing data), in six of them, at least one expert, generally an expert retrained by the prosecution, raised directly or indirectly the possibility of malingering. In comparison, the remaining 15 cases did not include any records of issues of malingering. The suspected malingering rate is not far from the 19% base rate of probable malingering and symptom exaggeration in criminal cases (Mittenberg et al., 2002) or the 18.5% rate found in previous CFE findings (Blume et al., 2014).

#### 3.3.3. Prior Adjudicative Competence Evaluation

Next, we explored whether the successful *Ford* claimants had a history of competency litigation in prior proceedings and, if so, the outcomes of those cases. Among the various types of criminal competencies, we focused on competency to stand trial (CST).^11^ Defined by the landmark Supreme Court case *Dusky v. United States* (1960) as the “sufficient present ability to consult with his lawyer with a reasonable degree of rational understanding,” CST remains the most commonly conducted competency evaluation in the United States today (Pirelli et al., 2011; Murrie et al., 2023). In the present study, among the successful *Ford* claimants with available CST information (*n* = 23; 5 cases with missing data), 18 claimants were deemed competent to stand trial at least once, while three claimants were deemed incompetent at least once. Two cases demonstrated a shift in competency status: one individual was initially deemed competent but later retroactively found incompetent, while another was first deemed incompetent but subsequently found competent. Notably, in one case where the individual was deemed competent to stand trial, this determination followed a second attempt, as the first resulted in a mistrial.

#### 3.3.4. Ford Claims History and Evaluators

We now focus on the outcomes and specific evaluation details of the CFE process itself. Among the 28 successful *Ford* claimants, all were declared incompetent for execution at least once in their CFE evaluation (the inclusion criterion). Notably, among claimants who had more than one CFE evaluation, five claimants were initially deemed competent but later found incompetent, while one was initially deemed incompetent but subsequently declared competent.

When examining the mental health professionals involved in the CFE evaluations for the successful *Ford* claimants, in 23 cases, more than two types of mental health professionals participated in the evaluations. The breakdown, as shown in Table 10, is as follows: psychiatrists (22 cases), psychologists (17 cases), neurologists (two cases), general practitioners (one case), counselors (one case), state hospital personnel (one case), and social workers (one case).

## 4. Discussion

Our study focused on the shared characteristics and experiences of a subset of individuals with SMIs on death row—specifically, those whose conditions have led to legal determinations that they are not competent to be executed. Several observations emerged after closely reviewing the successful *Ford* claimants, which we argue are deeply intertwined with their mental conditions. We reiterate our earlier caveat that the existing data are too limited to conduct multivariate analyses, so the findings should be regarded as suggestive—intended to spur the research questions—rather than as definitive.

### 4.1. Characteristics of Successful Ford Claimants

Although our limited sample size precludes broad generalizations, our findings illuminate several distinctive characteristics among successful *Ford* claimants that merit further scholarly attention.

#### 4.1.1. Advanced Age and Prolonged Confinement

The demographic profile of these claimants is notably advanced: predominantly older adults. Furthermore, the average duration of incarceration on death row is over thirty years. This is not surprising given that a *Ford* CFE claim cannot be raised in most jurisdictions until the individual has exhausted their other appeals (which generally take years) and an execution date is imminent. This prolonged confinement, under conditions that in many places are similar to solitary confinement, may exacerbate existing mental health challenges and contribute to a persistent state of legal limbo.

#### 4.1.2. Racial Composition

The claimant population is racially diverse. Black individuals comprise the largest segment, followed by White individuals, with smaller proportions of Latino, Native American, Mixed Race, and other classifications. Preliminary comparisons suggest that White and Latino claimants may be underrepresented relative to their prevalence within the broader death row population. Although it remains uncertain whether these disparities stem from the relatively small number of successful *Ford* claimants or reflect underlying systemic factors, this observation underscores the need for further investigation into the influence of race on legal outcomes and competency evaluations for individuals with SMIs.

#### 4.1.3. Educational Attainment

None of the claimants attained a college degree, and many exhibit low overall educational achievement. This observation raises questions about the potential interplay between limited educational opportunities and the challenges associated with SMIs. Future research should explore whether these educational disparities intertwine with adverse legal and mental health outcomes.

#### 4.1.4. SMIs, Psychiatric Comorbidity, and Schizophrenia

Not surprisingly, the prevalence of SMIs and psychiatric comorbidities is remarkably high in this group. Many claimants experienced the onset of their mental illnesses prior to incarceration and received inadequate treatment during confinement. Notably, the incidence of schizophrenia is quite high, suggesting that the chronic, debilitating nature of this disorder—compounded by the adverse conditions of death row—may be closely associated with determinations of legal incompetence for execution.

In summary, while our dataset limits the generalizability of these observations, the unique profile of successful *Ford* claimants—characterized by advanced age, limited educational attainment, high rates of SMIs (particularly schizophrenia), and the potential detrimental effects of extended incarceration—warrants further investigation into the intersections of mental health, education, and legal outcomes in the context of death row sentencing.

### 4.2. Successful Ford Claimants’ Crime of Convictions

#### 4.2.1. Temporal Influences

An examination of the temporal dimensions of successful *Ford* cases reveals noteworthy trends. Our findings suggest that prolonged incarceration might be associated with a progressive decline in mental health—a phenomenon often referred to as death row syndrome (Harrison & Tamony, 2010). In this context, inmates with earlier offenses are more likely adjudicated as incompetent for execution, reflecting the cumulative psychological impact of extended death row confinement. Consequently, if this pattern persists, individuals sentenced in the 1990s and early 21st century may increasingly file *Ford* claims, underscoring the long-term effects of incarceration on mental health.

#### 4.2.2. Geographical Influences

Geographical factors further complicate the landscape of successful *Ford* claims. Consistent with the previous literature (Blume et al., 2014), our initial observations indicate that the overall number of successful *Ford* cases is low across states that impose the death penalty, creating a floor effect that limits the potential for robust cross-state comparisons. Nonetheless, notable variations in the rate of successful *Ford* claims emerge when these figures are considered relative to the number of death row inmates and executions in each state. The underlying causes of these discrepancies remain unclear, warranting further investigation. Future research should explore whether the adoption of unified CFE standards might address these state-level variations, particularly given the ambiguity of existing Supreme Court guidelines. Moreover, the ratio of successful *Ford* cases may not fully capture the true extent of severe mental illness among death row inmates in each state, an issue that requires additional data to resolve.

#### 4.2.3. Victim Demographics and Defendant–Victim Relationship Dynamics

Our analysis indicates a pattern that the victim profiles in successful *Ford* cases diverge from those typically observed in U.S. homicide cases, suggesting that the dynamics of these offenses may be influenced by SMIs.

**Number of Victims.** Successful *Ford* cases are characterized by a higher incidence of multiple-victim incidents, resulting in a greater average number of fatalities per case compared with general homicide trends. This elevated multiplicity points to the severe and often atypical nature of these offenses.

**Victim Gender.** While general male-on-male homicides often involve young adult victims—predominantly in intraracial conflicts linked to gang activity and firearms—the pattern in these cases is notably different. Although all perpetrators in our sample are male, the crimes do not conform to the typical gendered dynamics of violence. Incidents involving female victims in these cases do not stem from domestic or sexually motivated crimes; rather, they tend to arise in contexts that deviate from conventional patterns, suggesting an alternative underlying dynamic.

**Victim Race.** Traditional homicide data indicate that male victims are often young men of color involved in intraracial violence, as well as female victims involved in intraracial domestic or sexually motivated crimes. In contrast, the racial composition of victims in successful *Ford* cases is more heterogeneous, including evidence of transracial victimization. This broader racial spectrum further distinguishes these cases from conventional homicide patterns and implies a different set of influencing factors.

**Defendant–Victim Relationship.** A striking finding is the nature of the relationship between defendants and their victims. In these cases, there is a disproportionately high number of male family members and female non-family members among the victims, as well as child victims (especially those under the age of 12) featuring prominently. In more than half of the child victim instances, the victims are either the claimants’ children or other family members. This pattern contrasts sharply with general homicide cases, where male family members and female non-family are less frequently targeted, and suggests that the underlying motivations in successful *Ford* cases may be linked to the cognitive and behavioral disruptions associated with SMIs.

The confluence of a higher number of victims per incident, atypical gender and racial victim profiles, and unusual defendant–victim relationships indicates that the dynamics of these offenses may be driven by factors distinct from those underlying general homicides. Instead, these patterns suggest that the presence of SMI might play a critical role in shaping both the selection of victims and the nature of the crimes committed. Further research, with a larger dataset, is necessary to confirm these preliminary findings and to elucidate the complex interplay between mental health and homicide dynamics.

### 4.3. Professional Considerations of Successful Ford Claims

Again, we emphasize that our observations are derived from a small sample size and may evolve as additional case data become available; however, some patterns are worth noting for further observation.

#### 4.3.1. Inconsistent Presentation of Mental Health Evidence

Mental health evidence was presented inconsistently across legal stages—appearing in about two-thirds of trials and PCR phases, as well as nearly all habeas corpus proceedings. This variability may stem from insufficient awareness, inadequate training, resource constraints, or ineffective counsel, contrary to expectations that mental health mitigating factors would be central to these cases.

#### 4.3.2. Prior Competency Evaluations

Only a small percentage of successful *Ford* claimants had previously been deemed incompetent to stand trial, a figure comparable to earlier findings (Blume et al., 2014). Despite the severity of their mental illnesses at trial, all claimants were ultimately found incompetent for execution, highlighting a potential disconnect between their actual mental state and the outcomes of competency assessments.

#### 4.3.3. CFE Evaluations and Evolving Standards

Our review of CFE evaluations reveals an evolving process that may indicate either a degree of arbitrariness or a changing dynamic in the clinical profiles of claimants over time. These changes complicate the interpretation of mental health evidence and suggest that the standards for evaluation might not be consistently applied.

#### 4.3.4. Psychiatric Involvement and Interdisciplinary Evaluation

Notably, a number of cases lacked any involvement of psychiatrists despite their recognized expertise in diagnosing mental illness. Although a diverse range of professionals participated in many evaluations, the absence of psychiatric input in a significant number of cases raises concerns about the accuracy of these assessments and underscores the need for a more comprehensive interdisciplinary approach.

Collectively, these findings underscore a potential gap between the documented mental status of the claimants, the evidence presented during legal proceedings, and the conclusions reached by evaluators. The low rate of mental health evidence at trial, inconsistencies in competency assessments, and the lack of consistent psychiatric involvement call for enhanced resources, improved training, and standardized protocols. Future research should address these discrepancies to ensure that evaluations more accurately reflect the realities of SMIs among this unique population.

## 5. Conclusions

In this study, we provided the first comprehensive analysis of all successful *Ford* claimants, a small yet important subset of persons on death row deemed incompetent for execution within the U.S. legal system. These individuals, found incompetent for execution, embody the intersection of SMIs and the death penalty. Despite being spared execution, their prolonged suffering—often exacerbated by systemic failures—reveals shortcomings within the criminal justice system. The claimants, with a pattern of being severely mentally ill, undereducated, and White and Latinx are underrepresented, have spent an average of over 30 years on death row with minimal access to treatment. Nearly all were diagnosed with schizophrenia, with four out of five experiencing co-occurring SMIs, often with early onset before their offense. Furthermore, many of the crimes committed by these individuals lack discernible motives or involve motives that defy rational understanding. The victims of these crimes, frequently male family members, female non-family members, or children, and the atypical nature of these cases highlight the troubling reality of puzzling dynamics, such as multiple victims, child victims, transracial homicides, non-violent male-on-male homicides, and non-sexual femicides, which are arguably linked to mental illness.

This study also exposed significant procedural deficiencies, including a lack of mental health evidence in at least a third of cases at trial and PCR phases, with evidence of the person’s SMIs only emerging at the habeas corpus stage. Competency to stand trial challenges were frequently denied, and not all cases included psychiatric evaluations at critical stages. These inconsistencies, combined with state-by-state disparities, point to the marginalization of mentally ill individuals within the legal process, raising both ethical and procedural questions.

If corroborated with larger datasets that permit multivariate analyses, these findings suggest a need for systemic reforms to address the unique vulnerabilities of this population. Notwithstanding the small number of successful claimants that we examined here, the death row population continues to grow older, raising the specter of increased deterioration of mental health over time. If true, this raises ethical and legal concerns regarding the execution of individuals with SMIs. It is crucial that the legal system implements more robust safeguards, including timely access to mental health treatment and comprehensive evaluations throughout the trial process, to prevent further injustices. The recent advocacy for categorical bans on executing individuals with specific mental illnesses must be part of a broader, more psychiatrically-informed approach to criminal justice.

We acknowledge that this study is not without limitations. As we have repeated throughout, the small number of successful *Ford* claimants restricts the generalizability of our findings, and further research with larger, more diverse samples is essential to validate and expand on these conclusions using multivariate analyses with all relevant moderators in the model, something beyond the capability of the present data. Future studies should explore the intersection of mental illness and the death penalty in greater depth, considering broader populations and the systemic factors that contribute to the execution of mentally ill defendants. Additionally, a more nuanced understanding of the ethical and procedural dimensions of these CFE cases could inform reforms that ensure fairness and compassion in legal proceedings.

Ultimately, this research serves as a call for a more informed approach to capital punishment, one that prioritizes justice and human dignity. The study of successful competency-to-be-executed cases is a vital step in ensuring that legal decisions consider not only the facts of a crime but also the complex mental health issues at play. Moving forward, it is important that the criminal justice system recognizes and addresses the unique challenges faced by mentally ill individuals on death row, ensuring that justice is both fair and humane.

## Figures and Tables

**Table 1:** Number of death-sentenced cases, executions, and ford claims from 1986 to 2013.

Case	*n*
Death-Sentenced Cases *	5724
Executions **	1280
*Ford* Claims	141
*Ford* Claims on the Merits	92
Unsuccessful *Ford* Claims	120
Unsuccessful *Ford* Claims on the Merits	71
Unsuccessful *Ford* Claims on the Procedural Grounds	49
Successful *Ford* Claims	21

Note. This table is adapted from Blume et al. (2014)’s Table 1. According to the original note: * The number represents the number of death sentences issued from 1 January 1986 through 31 December 2012. This number includes some sentences imposed in 1986 prior to the decision in *Ford v. Wainwright* on 26 June 1986; however, the available data is not disaggregated by month. ** The number represents the number of executions carried out since the *Ford* decision through 28 July 2013.

**Table 2:** Serious mental illness (SMI) and intellectual disability in successful *Ford* cases compared with the U.S. jail/prison populations and the general population.

	Successful *Ford* Claimants	*U.S. Incarcerated Population*	U.S. General Population
Serious Mental Illness			
Schizophrenia	86%	2–6.5%	0.33–0.48%
Personality Disorders	39%	21%	9.1%
Substance Use Disorders	29%	30%	6.6–13.2%
Depressive Disorders	29%	36.9%	8.3%
Overall SMI	100%	10–16.7%	6%
Intellectual Disability	21%	10%	1.65%

**Table 3:** Comorbidity patterns in successful *Ford* cases.

Case No.	Schizophrenia	Substance Use Disorder	Personality Disorder	Other SMIs
1	+	−	+	+
2	+	+	−	+
3	+	+	−	+
4	+	+	+	+
5	+	−	+	+
6	+	−	−	+
7	+	+	+	−
8	+	−	+	+
9	+	−	+	+
10	+	−	+	+
11	+	+	−	+
12	+	−	+	+
13	+	+	−	+
14	+	−	+	+
15	+	+	−	+
16	+	−	−	+
17	+	−	+	−
18	−	+	−	+
19	+	−	−	+
20	+	−	−	+
21	+	−	−	−
22	+	−	+	−

*Note.* + indicates the presence of an SMI, while − indicates its absence. The case numbers in the table do not convey any specific meaning beyond this, and the case numbers are not relevant to the other tables in this article. Our goal was to present a general trend in the comorbidity patterns without revealing identifiable information.

**Table 4:** Successful *Ford* cases by state in states retaining the death penalty.

State	*n*	State	*n*	State	*n*
Alabama	0	Kentucky	0	Oregon *	0
Arizona	1	Louisiana	2	Oklahoma	3
Arkansas	2	Mississippi	1	Pennsylvania *	2
California *	1	Missouri	1	South Carolina	2
Florida	0	Montana	0	South Dakota	0
Georgia	0	Nebraska	0	Tennessee *	0
Idaho	2	Nevada	0	Texas	9
Indiana	0	North Carolina	1	Utah	0
Kansas	0	Ohio *	1	Wyoming	0

*Note.* * denotes the states that retain the death penalty but have paused executions through executive action.

**Table 5:** Comparison of the number of successful *Ford* cases and the number of prisoners on death row by state in states that have ever ruled successful *Ford* claims.

State	Successful *Ford* Cases *n*	Prisoners on Death Row *n*	Ratio of Successful *Ford* Cases to Death Row Inmates %
Arizona	1	114	0.9%
Arkansas	2	28	7.1%
California	1	665	0.2%
Idaho	2	8	25.0%
Louisiana	2	63	3.2%
Mississippi	1	36	2.8%
Missouri	1	18	5.6%
North Carolina	1	140	0.7%
Ohio	1	129	0.8%
Oklahoma	3	40	7.5%
Pennsylvania	2	123	1.6%
South Carolina	2	36	5.6%
Texas	9	192	4.7%

*Note.* The latest reported data on the number of prisoners on death row across states, sourced from the Death Penalty Information Center (2023), are used as a baseline to illustrate the size of death row populations across states.

**Table 6:** Comparison of the number of successful *Ford* cases and the number of executions by state in states that have ever ruled successful *Ford* claims.

State	Successful *Ford* Cases *n*	Executions *n*	Ratio of Successful *Ford* Cases to Executions %
Arizona	1	40	2.5%
Arkansas	2	31	6.5%
California	1	13	7.7%
Idaho	2	3	66.7%
Louisiana	2	28	7.1%
Mississippi	1	23	4.3%
Missouri	1	99	1.0%
North Carolina	1	43	2.3%
Ohio	1	56	1.8%
Oklahoma	3	124	2.4%
Pennsylvania	2	3	66.7%
South Carolina	2	43	4.7%
Texas	9	587	1.5%

*Note.* The latest reported data on the number of executions since 1976 across states, sourced from the Death Penalty Information Center (2023), are used as a baseline to illustrate the size of executions across states.

**Table 7:** Offender–victim relationship by race in successful *Ford* cases vs. U.S. homicide cases: a focus on black and white racial dynamics.

	Race		Successful *Ford* Cases	U.S. Homicide Cases
	Offender	Victim		
Intraracial	Black	Black	0%	89%
	White	White	18%	79%
Transracial	Black	White	36%	17%
	White	Black	5%	8%

*Note.* We calculated the ratios using different population numbers. For the present study, we used the total number of available cases with the race of the claimant (offender) and the victim, which amounted to 22 cases. For the U.S. homicide cases, we used the total number of Black deaths to calculate Black-on-Black and White-on-Black homicides and the total number of White deaths to calculate White-on-White and Black-on-White homicides.

**Table 8:** Offender–victim relationship by gender in successful *Ford* cases vs. U.S. homicide cases.

	Successful *Ford* Cases		U.S. Homicide Cases	
	Victim Gender			
	Female	Male	Female	Male
Non-Stranger				
Family Member	28%	24%	50%	16%
Non-Family Member	40%	18%	26%	40%
Stranger	28%	44%	12%	21%
Unknown	4%	0%	20%	33%

**Table 9:** Successful *Ford* cases by mental health evidence presentation across phases of review of capital cases.

Phase	Mental Health Evidence *n* (%)		
	Present	Absent	Missing Data
Trial	17 (61%)	7 (25%)	4 (14%)
Post-Conviction Relief	18 (64%)	1 (4%)	9 (32%)
Habeas Corpus	26 (93%)	0 (0%)	2 (7%)

**Table 10:** Mental health professionals involved in CFE evaluations of successful *Ford* cases.

Case No.	Mental Health Professionals		
	Psychiatrists *n*	Psychologists *n*; Details	Others *n*; Details
1	5	-	-
2	2	1	-
3	6	1; Unlicensed	-
4	>1	1	-
5	1	1	-
6	2	1	-
7	-	-	1; State Hospital Personnel
8	1	1	1; Social Worker
9	1	-	1; Neurologist
10	1	-	-
11	1	-	-
12	1	2	-
13	1	1	-
14	-	-	2; General Practitioners
15	>1	-	-
16	>1	>1	-
17	1	-	1; Counselor
18	-	-	1; Psychology Expert
19	1	1	>1; Mental Health Professional
20	3	1	-
21	-	-	1; Neurologist
22	1	-	-
23	1	-	-
24	1	1; Forensic Psychologist	-
		1; Clinical Psychologist	
		1; Psychologist (Ed.D.)	
25	1	1	-
26	-	-	-
27	1	1	-
28	-	1	-

*Note.* The case numbers in the table are not intended to convey any specific meaning beyond this context and are irrelevant to the other tables in this article. Our goal is to highlight the general trends in the types of mental health professionals involved in the CFE evaluation process while ensuring that no identifiable information is disclosed.

## Data Availability

The de-identified raw data supporting the conclusions of this article will be made available by the corresponding author upon request.

## Notes

1 To provide context on why we used a networking approach to solicit the names of successful *Ford* claimants: Prior research—including a study conducted by our second author (Blume et al., 2014)—has established that *Ford* claims are filed under highly rare circumstances, even within the realm of capital defense. In most cases, such claims are only pursued when an execution date has been set. A national email group connects professionals—including scholars, attorneys, and NPO/NGO representatives—who closely monitor capital sentencing and executions in the United States. This group regularly exchanges litigation strategies, making it relatively easy to track legal representation, execution date orders, *Ford* claim filings, and claim outcomes. If a *Ford* claim is granted, the client will, in most cases, avoid execution, and other members of the email chain are typically aware of the outcome. Given this structure, obtaining information on recent *Ford* claim filings from our second author—who is a member of this professional network—was a logical and effective approach. Once we received responses from the email chain, we systematically compared the information with the known cases identified by Blume et al. (2014), Perlin and Harmon (2021), and Perlin et al. (2022, 2023) and confirmed their inclusion through Westlaw Edge and Lexis Advance legal database searches. This process ensured that each identified case met our criteria while allowing us to exclude cases with unsuccessful or “unripe” *Ford* claims.

2 While operational definitions of SMI vary (Gonzales et al., 2022), they are broadly understood as a “mental, behavioral, or emotional disorder resulting in serious functional impairment that substantially interferes with or limits one or more major life activities” (American Psychiatric Association, 2022; National Institute of Mental Health, 2024). Common examples of SMIs include major depressive disorder, schizophrenia, bipolar disorder, obsessive-compulsive disorder, panic disorder, post-traumatic stress disorder, and borderline personality disorder.

3 Schizophrenia “has a complex presentation with a multifactorial cause” (McCutcheon et al., 2020). The latest diagnostic criteria for schizophrenia, as outlined in the Diagnostic and Statistical Manual of Mental Disorders, Fifth Edition (DSM-5), highlight the major symptoms of the disorder, which include delusions, hallucinations, and/or disorganized speech. According to the American Psychiatric Association (American Psychiatric Association, 2024b), schizophrenia is characterized as a “chronic brain disorder” with positive symptoms (e.g., hallucinations), negative symptoms (e.g., affective flattening, alogia, asociality, avolition, and anhedonia), and disorganized symptoms (e.g., confused thinking, disordered speech, difficulty with logical reasoning, and occasionally bizarre behavior or abnormal movements). Additionally, schizophrenia is marked by episodes during which individuals may be “unable to distinguish between real and unreal experiences”. Similarly, the National Institute of Mental Health (n.d.-b) notes that while “the course of schizophrenia varies among individuals,” it is “typically persistent and can be both severe and disabling”.

4 According to the American Psychiatric Association (2024c), “Substance use disorder (SUD) is a complex condition characterized by the uncontrolled use of a substance despite harmful consequences. Individuals with SUD exhibit an intense focus—sometimes referred to as an addiction—on using a particular substance or substances, such as alcohol, tobacco, or other psychoactive substances, to the extent that their ability to function in daily life becomes impaired”. The latest diagnostic criteria for SUD, as outlined in the DSM-5, represent a significant revision from previous editions (Hasin et al., 2013), classifies SUD under substance-related and addictive disorders and includes disorders related to ten separate classes of substances: alcohol, caffeine, cannabis, hallucinogens, inhalants, opioids, sedatives, hypnotics or anxiolytics, stimulants (including amphetamine-type substances, cocaine, and other stimulants), and tobacco.

5 According to the American Psychiatric Association (2024d), “Personality disorders are long-term patterns of behavior and inner experiences that differ significantly from what is expected. They affect at least two of the following areas: ways of thinking about oneself and others, ways of responding emotionally, ways of relating to other people, and ways of controlling one’s behavior”. Similarly, the National Institute of Mental Health (n.d.-a), citing the DSM-5, describes personality disorders as “an enduring pattern of inner experience and behavior that deviates markedly from the expectations of the individual’s culture,” noting that “these patterns tend to be fixed and consistent across situations and lead to distress or impairment”. The DSM-5 currently identifies ten types of personality disorders: paranoid, schizoid, schizotypal, antisocial, borderline, histrionic, narcissistic, avoidant, dependent, and obsessive-compulsive.

6 The neurocognitive disorders encompass cases historically referred to as mild organic brain disorder, organic brain dysfunction, organic brain damage, organic brain syndrome, or brain injury, reflecting changes in diagnostic terminology over time.

7 Intellectual disability is characterized by “significant limitations in both intellectual functioning and adaptive behavior” (American Psychiatric Association, 2024a). According to the American Psychiatric Association, “cognitive or intellectual functioning has traditionally been measured through intelligence quotient (IQ) tests, with an IQ of less than 70 recommended for a clinical diagnosis of intellectual disability”. and “adaptive functioning is assessed through standardized measures, along with interviews with individuals such as family members, teachers, and caregivers”.

8 Although it is valid to suggest that cases involving female victims are more likely to result in the death penalty (Holcomb et al., 2004; Williams et al., 2007; Royer et al., 2014), which could help explain the difference between the successful *Ford* cases and the national pool of homicide data, it is also important to consider that these cases may present a distinct picture of gendered homicide. Factors such as the presence of sexual victimization, the method of killing, the relationship between the victim and the defendant, and whether the victim had family responsibilities (Royer et al., 2014) may differentiate these cases from other homicide cases, warranting further exploration.

9 Similar to the previous footnote, although it has long been found that victim race plays a role in the death penalty—particularly with a racial disparity in killings of whites versus blacks, where killings of whites are more likely to result in the death penalty (Radelet, 1981; Holcomb et al., 2004; Ulmer et al., 2020) and, conversely, the ‘black male victim effect’ (Girgenti, 2015) is associated with being deemed less cruel and less likely to result in the death penalty—our database shows that the number of white victims is similar across the successful *Ford* cases and the national homicide pool. However, there may still be potential differences in the dynamics at play, suggesting that these killings could differ from the national pool of homicide cases.

10 The trial phase involves the initial proceedings, including determining guilt or innocence, followed by sentencing and direct appeal. If a defendant is convicted and sentenced to death, and the convictions and sentence are affirmed on the first (direct) appeal, the PCR phase allows for challenges to the conviction or sentence in state courts based on claims of legal errors or new evidence. The federal habeas corpus phase provides an opportunity, albeit limited in recent decades by Congress and Supreme Court rulings limiting a federal court’s ability to overturn a state court conviction or death sentence, for persons on death row to ask federal courts to review claims of constitutional error. In theory, together, these phases are intended to ensure a comprehensive review of capital cases, protecting defendants’ rights and upholding the fairness of the judicial system, although there are a number of documented cases where severe injustices were not rectified by state and federal courts in the appeals process.

11 We also examined whether the successful *Ford* claimants filed for other competency challenges, such as competency to plead guilty and competency to waive counsel. Unfortunately, due to a substantial portion of missing data, we could not report meaningful results. However, the following findings emerged. In terms of competency to plead guilty, among the successful *Ford* claimants, 16 had missing data. Only one individual was ever deemed competent to plead guilty. Notably, in 14 cases, a competency evaluation to plead guilty was not conducted—either because it was not applicable to the case or due to unwillingness. In terms of competency to waive counsel, of the successful *Ford* claimants, 17 had missing data. Only three individuals were deemed competent to waive counsel, while one case involved a claimant being deemed incompetent to waive counsel on at least two occasions. Additionally, in a subset of seven cases, no competency evaluation to waive counsel was conducted, primarily due to unwillingness.
